# Supplementary material for: A centrifugation-based physicochemical characterization method for the interaction between proteins and nanoparticles
Source: Nat Commun. 2016 Oct 20;7:13121. doi: 10.1038/ncomms13121 (PMC5080432; doi:10.1038/ncomms13121)
Supplement: Supplementary Information — Supplementary Figures 1-6, Supplementary Table 1 and Supplementary Note 1. [file ncomms13121-s1.pdf]

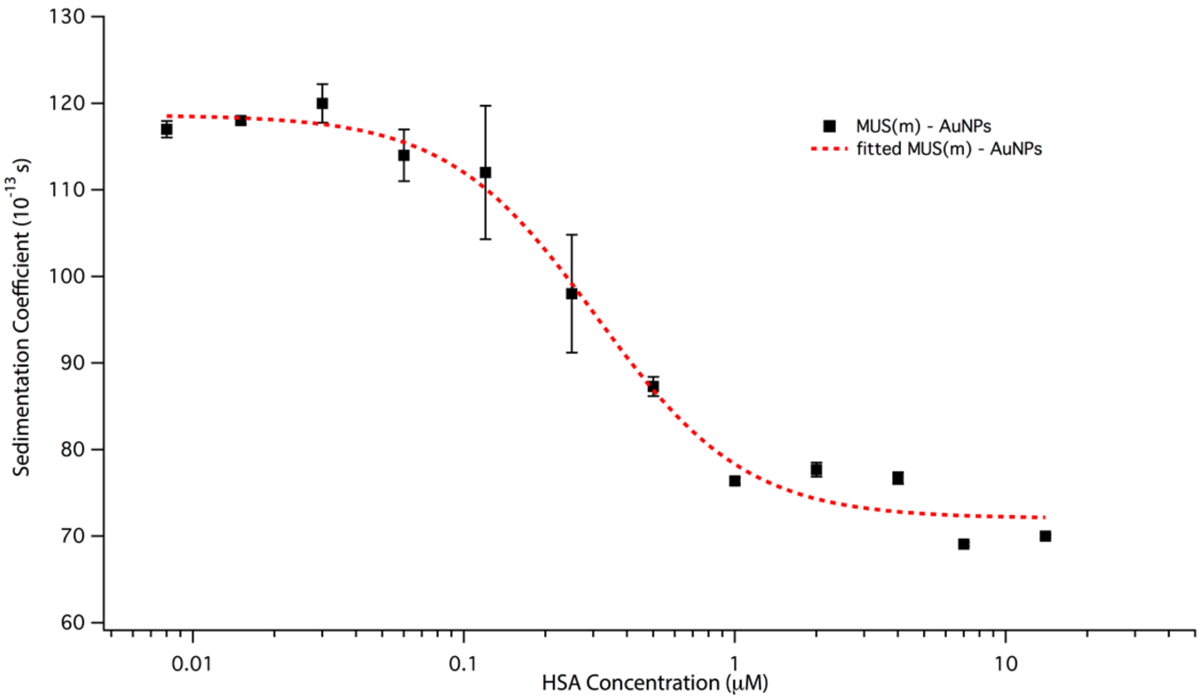


**Supplementary Figure 1.** Adsorption isotherm for MUS(m)-AuNPs – HSA.

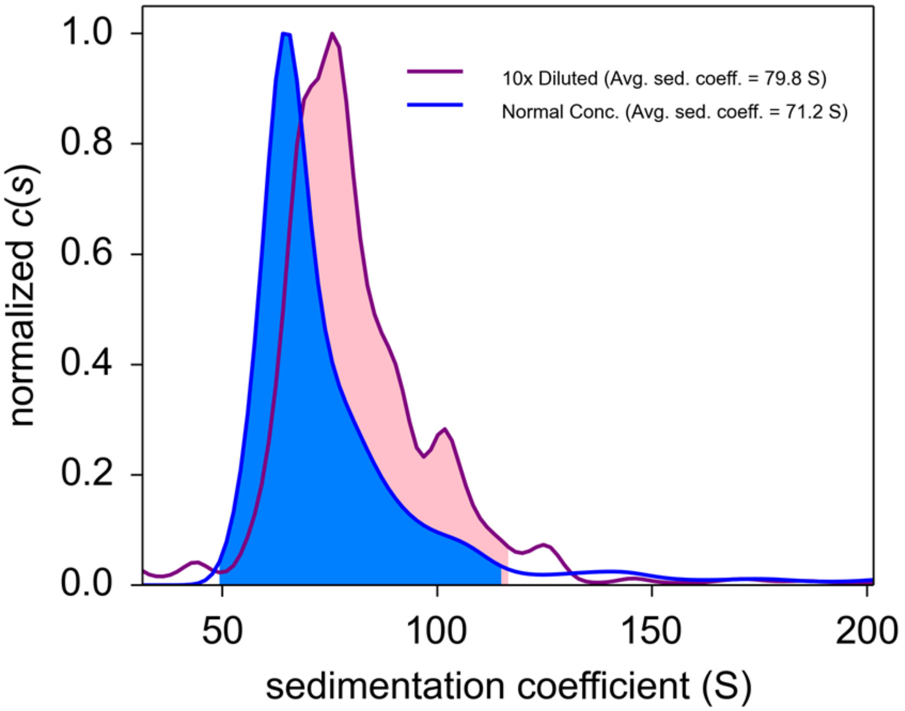


**Supplementary Figure 2. Reversibility Experiments using AUC:** MUS(m)-AuNPs ($d_H$ = 7.6 nm) is mixed with 1 $\mu$M BSA solution and incubated 16 hours at 20 °C. Then, a portion from this solution is taken and diluted 10 times with 10 mM KCl solution to another tube. The new diluted sample is left to re-equilibrate for additional 16 hours at 20 °C. Both solutions are measured in AUC with SV-experiment and *c(s)* distributions are compared. Figure shows the apparent shift in the *c(s)* distribution upon dilution which indicates the reversibility. Blue and pink areas represent the range where average sedimentation coefficient is calculated.

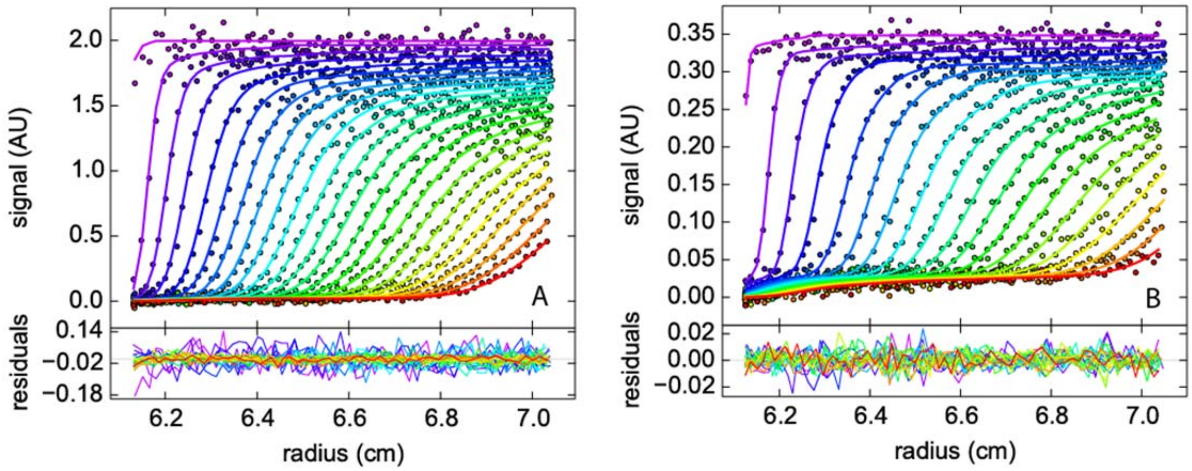


**Supplementary Figure 3.** Lamm equation fitted sedimentation profiles of (A) Normal and (B) 10 times diluted concetration of MUS(m)-AuNPs – BSA mixture.

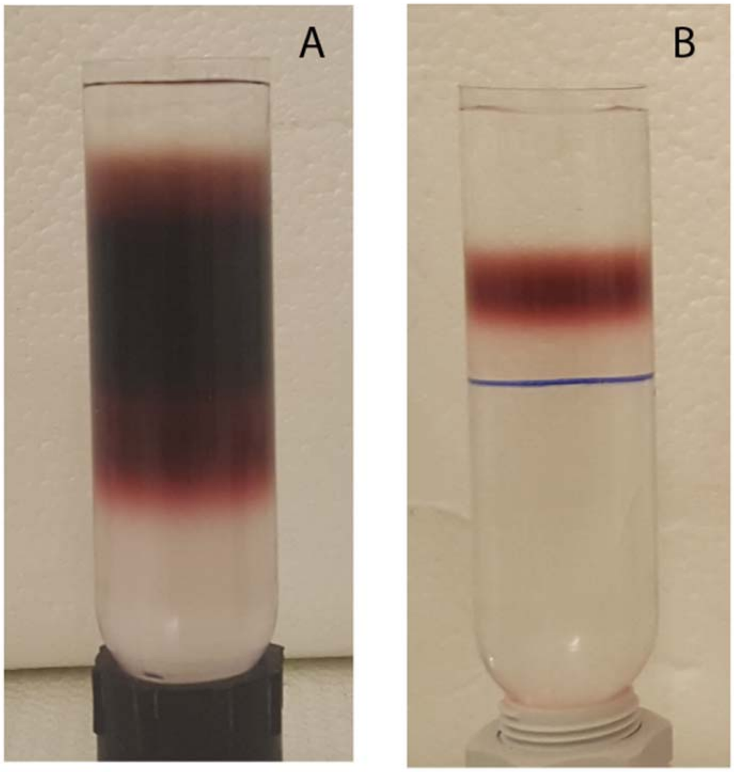


**Supplementary Figure 4.** MUS(m)-AuNPs centrifuged in continuous sucrose gradient (A) before and (B) after fractionation. Note the increased monodispersity judged by the spread of the nanoparticle zone after centrifugation.

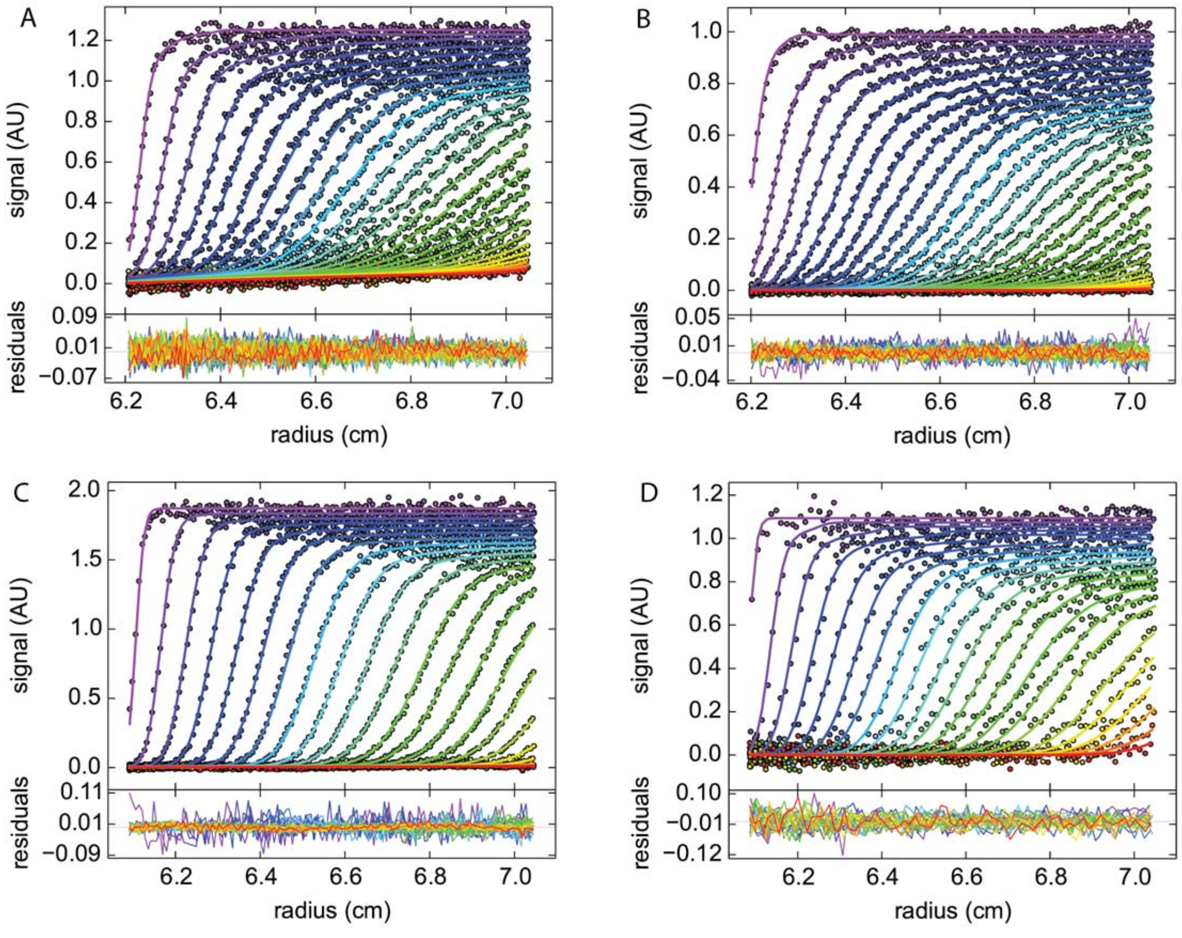


**Supplementary Figure 5:** AUC raw data (dots) with the Lamm equation fits (continuous lines) obtained with Sedfit Software. (A) MUS(s)-AuNPs, (B) MUS(m)-AuNPs, (C) MUA-AuNPs, (D) Citrate-AuNPs. Fitting residuals are in the bottom insets and show no systematic errors.

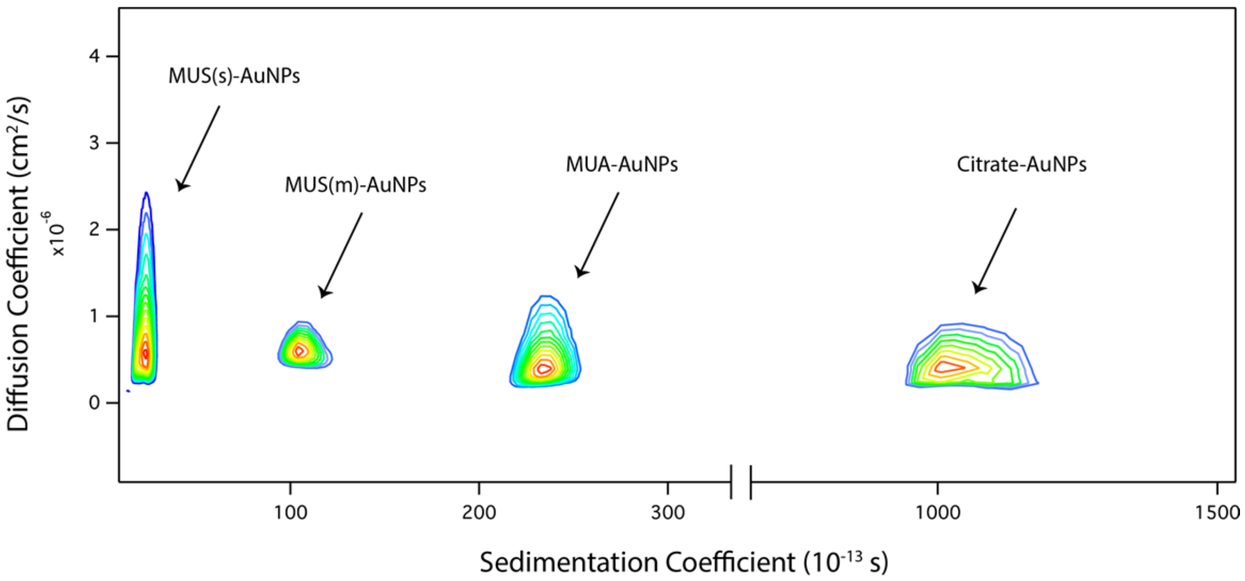


**Supplementary Figure 6: 2D c(s,D) analysis of nanoparticles.** Nanoparticles used in this work were synthesized and size fractionated to obtain monodisperse nanoparticles samples. Monodispersity was obtained with respect to the sedimentation coefficients. The plot shown here is the overlay of four separate experiments resulting in the shown contour plots.

| | Core Diameter (nm) | ~Mw $(g.mol^{-1})*10^{3}$ | ~Concentration Used $(mg.mL^{-1})$ | ~Concentration $(mol.L^{-1})*10^{-9}$ |
|---|---|---|---|---|
| MUS(s)-AuNPs | 2.2 | 82 | 0.01 | 120 |
| MUS(m)-AuNPs | 4.4 | 570 | 0.019 | 33 |
| MUA-AuNPs | 6.1 | 1400 | 0.036 | 26 |
| Citrate-AuNPs | 12.6 | 10000 | 0.06 | 6 |

**Supplementary Table 1.** Approximate nanoparticle molecular weights and concentrations used in experiments. Estimation of molecular weight based on the core size of the nanoparticles is shown in Supplementary Note 1.

**Supplementary Note 1.**

Molecular weight of gold nanoparticles is approximately calculated with hard sphere model. That is, estimated as the sum of molecular weight of gold core and ligands in the shell:

$$M_{NP} = \frac{V_{core}\, n}{V_{unit\ cell}} M_{Au} + A d_l M_l \quad (1)$$

where

$M_{NP}$: Molecular weight of a nanoparticle

$V_{core}$: Volume of a gold core

$n$: Number of Au atoms in a unit cell

$V_{unit\ cell}$: Volum of a Au unit cell

$M_{Au}$: Molar mass of Au element

$A$: Total surface of a gold nanoparticle

$d_l$: Ligand packing density (Number of ligands per unit area) (approximately 4.5 ligands per $nm^2$)

$M_l$: Molecular weight of a ligand.
